# Application of Bayesian Methods to Exposure Assessment of Area Concentrations at a Rubber Factory

**DOI:** 10.3390/ijerph6020622

**Published:** 2009-02-11

**Authors:** Yonghua He, Youxin Liang, Hua Fu

**Affiliations:** School of Public Health, Fudan University /Box 288, No 130, Dong’an Road, Shanghai 200032, PR China

**Keywords:** Exposure assessment, Bayesian methods, area concentration

## Abstract

The present study estimated area concentrations of airborne benzene in several workshops using Bayesian methods based on available historical measurements. A rubber products factory utilizing benzene was investigated. Historical measurements of benzene concentrations, expert experiences, and deterministic modeling were utilized in a Bayesian Method to estimate area concentrations. Historical concentrations (n=124) were available with the geometric mean of 15.3 mg/m^3^. The geometric mean of the current field measurements on the workstations ranged from 0.7 to 89.0 mg/m^3^. One of the seven historical geometric means by work locations significantly differed from the field measurements for equivalent locations, but none of the geometric means of Bayesian estimates were significantly different from the field measurement results. The Bayesian methods based on the historical measurements appeared to be a useful tool for more closely estimating area concentrations shown by field data than that predicted only using historical measurements.

## 1. Introduction

Exposure assessment (EA) is often necessary for epidemiologic research [1]. EA methods are the foundation for establishing reasonable data for human dose-response (effect) relationships. Reliable EA is also needed to guide exposure control measures for workers exposed to significant health risks. Multiple EA approaches have been set up [2]. Furthermore, subjective assessments for reconstructing exposure have also been used [3], but most options are prone to a variety errors and biases [1,4]. Bayesian methods have been introduced for occupational EA in combination with mathematical modeling [4–9].

A Bayesian approach may utilize exposure measurements to update a “prior” constructed by a deterministic model, where the parameters were determined by expert opinion informed with available information and measurements. If *e* represents the physical parameter of interest (that is in our case airborne benzene area concentration), and the measurement process furnishes a number represented by *M*, then the Bayesian expression can be described by the following equation:

(1) $$P_{post}\;(e/M)=\frac{P_{o}\;(e)P_{L}\;(M/e)}{P(M)}$$

where, *P*_0(_*_e_*_)_ is the probability distribution of *e*, *P*_L_(*M/e*) is the likelihood function that given the true value *e*, the measurement *M* is observed, *P*(*M*) is the probability that the measurement *M* is observed, and *P*_post_(*e/M*) is the updated probability (or the posterior) that the exposure is *e* given that the measurement *M* is observed.

Maximum Allowable Concentration (MAC, the concentration that should not be exceeded at anytime) [10] has been used in China as the occupational health standard from the 1950s to 2002. The present study takes benzene as an example to assess exposure levels with Bayesian methods based on historical area concentration measurements taken to evaluate MAC compliance.

## 2. Study Factory and Methods

### 2.1. Study Factory

A state-owned large rubber products factory in Shanghai, China, was recruited for the study. The factory was founded in 1954, and now includes 11 workshops for producing a variety of rubber hoses. The automation levels varied in the workshops, but the main processes were as the following: 1) rubber was extruded from a machine in the form of an inner layer of a tube assembly; 2) cotton threads or steel filaments were woven tightly on the surface of the rubber inner layer to increase the strength of the hose; 3) benzene was applied on the hose surface to make it tacky so the outer rubber layer adhered on it; 4) the outer rubber layer was extruded and tightly covered the fabric and the inner layers; 5) hoses were heated for vulcanizing. Benzene was mainly used as the bonding solvent between layers in eight of the workshops. Natural ventilation via doors and windows was present and fans were installed in the wall and/or ceiling of the building, but no local mechanical exhaust ventilation system was available at any worksite.

### 2.2. Bayesian Analysis Plan

In brief, the basis of the present methodology is a hybrid Bayes statistical approach [6,8,9], with the prior using exposure distributions obtained from a probabilistic mode and with available historical measurements. The Bayesian calculation plan was as follows.

1. For mathematical modeling, parameters of evaporation surface area for pollutant production, air flow rate, work environment distance from source to workers, products and production made, working hours every year, and process technology were provided to a professional expert team. The experts were asked to provide their opinion on the distribution and errors of the parameters for the deterministic model. The resulting parameters from the expert review were used to predict air concentrations.
2. Using parameters based on the expertise and historical working conditions, Monte Carlo simulation methods and the mathematical model were used to create the joint probability distributions and run for each sampled input parameters. These were the “prior” distributions, which characterized the uncertainty in the model output.
3. The estimated variance of the available historical air concentration data (the “real world” observations) were applied to estimate the parameters for the Bayesian likelihood function, P_L_(M/e).
4. Using WinBugs software [9] and Bayes rules, the prior was updated with the historical data to generate the posterior probability distribution of air concentrations P_post_(e/M).

**Figure 1.**
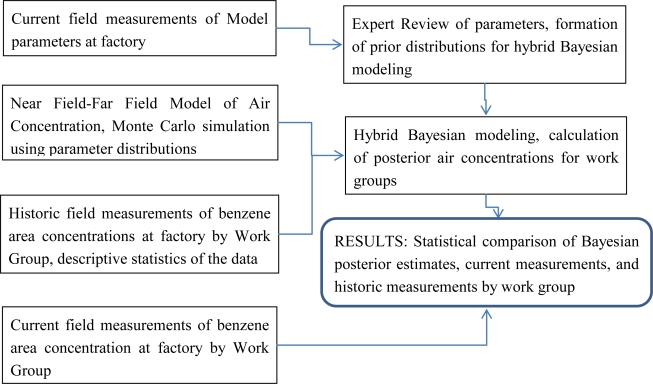
A Bayesian process chart.

A Bayesian process could be described as the following chart (Figure 1).

### 2.3. Data Collection and Worksite Investigation

The Shanghai Institute of Public Health and Supervision was visited and historical data of area benzene concentrations were collected. A spreadsheet was set up for coding the workstations at the factory and for abstracting information including work scheduling, raw materials used, work processes, job/task titles, and preventive measures. In the present study, most workshops changed production by changing the number of assistant workers and running hours, but the process and the product line number remained unchanged, consequently the area concentrations changed little. According to the field investigation and the hygienist experiences, we found that along with the traditional processes such as Slurry-making and Iron core tube assembly, several new and modern production lines were developed such as Line 1 to Line 3 where rubber hoses for automobile use were produced. Otherwise, there were no significant changes in work shift, raw materials used, work processes, or preventive measures prior to the current investigation. The annual productions of the workshops were provided by the factory, while the working hours were based on the recall of the workers who had been working there. A total of 15 similar exposure groups (SEGs) were created based on the processes, tasks and job activities, equipment and environment as well as area concentration data [11–13].

### 2.4. Data Selection for Deterministic Modeling

We employed three local experts in occupational health and industrial hygiene engineering to develop parameters for our EA methods, two of them were professors of occupational health and one was a professor of air conditioning and ventilation. They were provided with references about the goals of the study, literature about occupational EA, the processes of the factory, pictures of the workstation and job activities. Judging on feasibility and reliability, experts were invited to select the most appropriate deterministic model among Well-Mixed Box Model, Two-Zone Steady State Model and Eddy Diffusion Model to evaluate the workplace exposure. As a result, the Two-Zone Steady State Model [14] was selected based on the small working spaces, repetitive operation and poor ventilation. The model has relative simplicity while still accounting for variability in concentration with distance from the source. The choice was justifiable if several factors were met: 1) air in both the near- and far-field was adequately mixed; 2) a little air exchange was allowed; 3) and the pollutant production rate was stable. The near zone always means the hemisphere with the radius of the distance between the operator and the pollutant source. The model can be described by the following equation:

(2) $$C_{N,SS}=\frac{G}{Q}+\frac{2\text{G}}{S_{A}\cdot s}$$

where C_N_, _SS_: steady state concentrations of pollutant in the near zone (NZ), mg/m^3^; G: pollutant production rate, mg/min; Q: air flow rate supplied to the workshop, m^3^/min; S_A_: NZ free surface calculated using the distance from source to operator assuming hemisphere, m^2^; s: average of the random direction wind velocity across the near zone – far zone interface, m/min.

### 2.5. Current Worksite Benzene Air Sampling and Analysis

Benzene area samples were taken using sampling pumps (calibrated before and after sampling) and single section charcoal tubes of Chinese design (2nd Jianhu Electronic Instrument Factory of Jiangsu Province) at seven worksites where people were working, according to the current national standard (*Specifications of air sampling for hazardous substances monitoring in the workplace, GBZ159-2004*). The charcoal tubes have been widely used to collect airborne benzene in China for analysis with the NIOSH methods, and the results were reported to be comparable to those from NIOSH standard tubes [15]. Air samples were sealed and transported to the laboratory for analysis by gas chromatography according to NIOSH analytical method 1501. The air samples were stored at −20°C before analysis.

### 2.6. Estimation of Area Concentration with Bayesian Model

All input parameters including pollutant production rate, general indoor and NZ interface flow rate, for the selected model of the Slurry-making workshop and Cloth-lining workshop were assayed in the field. When the pollutant production was assayed, doors and windows were closed and wall ventilation fans were shut off. The sizes of the zones nearby the workstations varied, so it was not practicable to develop more detailed estimates of the pollutant productions. The workshop was divided into a sampling grid of 12 virtual spaces of equal area, with 12 air samplers distributed evenly in the grid, with samples taken for 10 min 6 times a day for 2 days. The virtual spaces in the Slurry applying workstation and Cloth-lining assembly workstation were 15 and 13 m^2^, respectively. The pollutant production rates were computed at the following formula:

(3) $$G=(C_{n}-C_{n-1})\times V/T$$

where G: the pollutant production rate, mg/min; C_n_: average concentration of the 12 area samplers at one round of sampling, mg/m^3^; C_n−1_: average concentration of the 12 area samplers at one round of sampling prior to C_n_, mg/m^3^; V: volume of the workshop, m^3^; T: sample timing between C_n_ and C_n−1_, min (=10).

The pollutant production rate data in the two workshops were normally distributed (verified with W-test). The arithmetic averages (AMs) of Slurry-applying workstation and Cloth-lining assembly workstation were 520 mg/min and 1,302 mg/min, respectively (Table 1).

Air velocities at the NZ interface and in the general workshop were assayed at 9:30 am, 12:30 pm, and 3:30 pm for 2 min on 2 days. The air velocity measurements were log-transformed and W-test was used to check the distribution of the log-transformed measurements. We found that the air velocities of both the NZ interface and the general workshop were log-normally distributed. Ventilation calculations were discussed below. The ventilation rates of the general room and the near field volume in the Slurry-applying workstation were 629 m^3^/min and 25 m^3^/min, respectively. They were 3,501 m^3^/min and 71 m^3^/min in Cloth-lining workstation, respectively (Table 1). It was obviously that ventilation varied between workstations and workshops because of the different free surfaces.

Most of the blueprints and records of the factory were not available, so layouts of the workshops were measured by the hygienist at the factory, and the data of the ventilation open surface, pollutant source, and the distance of pollutant to operator were measured in the field. As the Large water hose workshop did not exist any longer, the ventilation open surface, pollutant source, and the distance between the pollutant source and the operators in this workshop were estimated based on descriptions provided by the workers. Information on pollution sources, and surfaces emitting volatile organic solvents, work space volumes, and natural ventilation opening areas for all workstations were also collected and provided to the experts, and then the experts were asked to provide subjective judgment for each input parameter as a probability distribution for use with the mathematical model to construct the priors [6,16]. The historical process information for experts’ use in forming priors is listed in Table 2.

The experts agreed that the measurements of exhaust ventilation velocity data and the pollutant production rates of the two workstations should be considered as the anchoring information for the estimation. The pollutant production rate was normally distributed and proportional to the evaporating sources, as the ingredient of the solvents were the same. The experts also agreed that the area concentration, ventilation velocity (both workroom and the NZ interface) appeared to be log-normally distributed. The GM of air velocity of exhaust ventilation points obtained in the field was identified as air velocity for the calculation of ventilation volumes of all workshops. According to the field measurements, the GM of air velocity of general exhaust ventilation and the near field-far field zone interfaces were 0.61 m/s and 0.23 m/s, separately. The 90% confidence intervals (CI 90%) of the air velocity were between 0.2–5.0 times of the GM. The general ventilation volume for the workshop equaled one-half of the product of vented surface and the GM of the air velocity at the vent location.

The NZ interface surface was defined as a hemisphere, with the distance between the operators to the pollutant source as the radius. The pollutant production was proportional to the evaporating surface of pollutant source. According to the measurements of Slurry-applying and Cloth-lining workstation, the average pollutant production rate was 0.23 mg/cm^2^/min. Their CI 90% was 0.5–1.5 times of their averages. Priors of parameters provided by the experts were listed in Table 3. The Two-Zone Steady State Model was programmed with WinBugs. In the WinBugs code, the probability functions of pollutant production, air velocity and ventilation surface were coded as parameters for computation, and the historic measurements of the workstation were the observations. The model ran for 4000 iterations of input values by Monte Carlo sampling to obtain a simulated probability distribution of values for area concentrations of every workstation after burn-in of 1,000 iterations.

### 2.7. Quality Control

We had 20 duplicate samples in the Slurry-applying and Line 1–2 workstations, respectively, as representative workstations for low and high exposure levels. One set of the duplicates was sent to a U.S. laboratory for cross-checking. The results of a t-test on the duplicate samples showed they were statistically equivalent (data not listed). Data for the study were doubly entered into the computer system and automatically error-checked, with resolution of conflicting entries.

### 2.8. Statistical Analyses

The normal distribution of data was directly tested with W-test on the original data; the log-normal distribution was indirectly tested with W-test on the log-transformed data. The t-test was used to compare the means of historical measurements or Bayesian estimates with the current (collected as part of this study) worksite measurements. The *p* value was set at 0.05, double sided. All concentrations below the limit of detection (LOD) were replaced with LOD/[square root of 2] [17] for the computation of GM. All the statistical analyses were performed with SPSS 11.0.

## 3. Results

### 3.1. Benzene Levels by Current Field Survey

Area concentrations from the current field sampling (FM) were listed in Table 4. The GMs of the seven workstations’ data ranged from 0.7–89.5 mg/m^3^. Slurry-applying and Cloth-lining assembly had the highest benzene concentrations. Apart from the Steel weaving assistant workstation, the measurements of all workstations were log-normally distributed. The rate of samples below LOD was 17.4%.

During the period of the 1960s to 1984, samples were taken with a bubbler and analyzed by the digestive colorimetric method, with a LOD of 6 mg/m**^3^** [18]. From 1985 to 2003, samples were mainly taken by 1 min grab sampling with glass syringe and analyzed with gas chromatography, with a LOD of 0.6 mg/m**^3^**. Charcoal tube collection and gas chromatography analysis came into use in 2002, with a LOD 0.2 mg/m^3^[19].

We had 124 historical measurements (HM) of area concentration of benzene during the period of 1964 to 2003. The rate of samples below LOD was 13.7%, and the GM was 15.2 mg/m^3^. The historical measurements showed a big variation, e.g., 70% of the log-transformed standard deviations for measurements sampled within 17 years were greater than 3.

The GMs of the historical monitoring data from 15 workstations were 0.9–409.4 mg/m^3^ (Table 4). Six out of the 15 workstation HM were higher than that of the correspondent FM, but they were within their corresponding CI 95% of FM. There was no significant difference between the GMs of HM and FM except for 1 workstation. Two GMs of the HM were outside of the CI 90% of the FM. The ratios of the GMs of HM to that of the FM varied from 0.9 to 4.6, with an average of 2.7 ±1.4.

### 3.2. Benzene Levels of the Historical Measurements

### 3.3. Benzene Estimates by the Bayesian Model

Based on Bayesian Model (BM), the GMs of 15 workstations ranged 0.5–125.7 mg/m^3^ (Table 4). Five GMs of the BM estimates from seven workstations were higher than that of the correspondent FM, but they were within their corresponding CI 95% of FM, and there was no significant difference. The average ratio of GMs of BM to that of FM was 1.47±0.76, which is much lower than that of the ratio of the HM to the FM. Furthermore the standard deviation of the average ratio of GMs of BM to the FM was smaller than that of HM to FM.

## 4. Discussion

The challenge facing exposure assessors is how to combine and interpret the diverse information, which may be incomplete or sometimes conflicting, so a structured synthesis of the occupational EA information is often needed. On one hand, the quality control of the historical measurements such as MAC frequently remains a problem [20–22]. As the majority of the historical measurements were based on MAC concept and short-term sampling, a hard effort needs to be devoted for a reasonable data interpretation. On the other hand, expert judgment base on the historical working condition is always prone to subjective opinion and difficult to validate. Consequently a reasonable approach is to use the measurements, expertise, and mathematical models together to estimate the exposure levels. Then, Bayesian statistics are recommended because of their ability to synthesize all the information and produce output as the posterior through Monte Carlo simulations [1,6–8,23]. After the operation of the Bayesian methods, the estimates were closer to the field measurements than the historical data were, as the average ratio of GMs of BM to that of the FM was 1.47±0.76, while the ratio of HM to the FM was 2.7 ±1.4. Furthermore, as shown in geometric standard deviation of the estimates by Bayesian Methods was about half of that using HM. Even for the workstation that had not longer existed while investigating (e.g. the Large water hose workshop), the exposure determinants could be probed and adjusted, and the exposure levels could be “predicted”, retrospectively. The closer estimates and the smaller deviations suggested that the BM would have utility in refining the data when historical MAC measurements are used for the exposure assessment.

Field investigation of the work conditions, production rates, and technology process and health protection measures used guaranteed that the field measurements were consistent with the airborne benzene MAC concentrations to a certain extend over the history, so the current field sampling in this study was considered as the “gold standard” because the data were obtained successively over a 10-day period with representative operations selected. As reported by Collins *et al*., a series of historical measurements of 4,213 personal benzene exposure samples from 1980–1993 were collected and the subsequent correlation analysis showed no significant different trends on the data in terms of their periods and job titles [13], implying that the historical data could be adjusted by the current field measurements and used for retrospectively predicting the exposure levels at the similar workstations and job titles. It was similar to the present study, showing only one workstation out of seven with the GM significant different with the field measurements.

There are two obvious limitations in the present study. First, the Two-Zone Steady State Model is a simple model and not able to adequately mirror the time and space-varying complexities of the actual work places. This may introduce bias. However, more complex models require more parameters, and that aspect may introduce even more limitations on available data and possibly other biases. Additional research and comparisons of alternative models would be needed to further resolve this potential dilemma. Secondly, the Bayesian estimates are subject to the influence of the historical measurements. Consequently it is necessary to carefully consider the quality of the historic data and exercise caution where the quality is uncertain.

## 5. Conclusions Using the Hybrid Bayes Statistical Approach [6,8,9]

The findings from the present study suggest that the Bayesian methods using the historical measurements are a useful tool for more estimating area concentrations of benzene in the workplace.

## Figures and Tables

**Table 1.** Parameters measured in the factory (90 %CI).

Code	Workstation	Parameter	Number of measurements	**AM** or *GM*	Minimum	Maximum
1.1	Slurry applying	Pollutant production rate	12	**520 (515–524)**	353	1172
		General room ventilation	6	*629 (16–844)*	517	813
		NF interface ventilation	6	*25 (2.6–33.4)*	20	28.7
8.1	Cloth-lining assembly	Pollutant production rate	12	**1302 (1293–1311)**	698	3903
		General room ventilation	6	*3501 (30–19832)*	210	29207
		NF interface ventilation	6	*71 (1–339)*	16	420

Pollutant production rate, mg/min, Arithmetic Mean and 90 %CI

Ventilation volume, m^3^/min, *Geometric Mean and 90 %CI*

**Table 2.** Information for experts to estimate priors.

Code	Workstation	Tasks	Workshop ventilation surface, m^2^	Pollutant source	Distance between source to operator, m
1.1	Slurry making	slurry making	4.2	4 mixer machines, the volatile surface was about 870 cm^2^	0.5
2.1	Core assembly	similar with the cloth-lining assembly	20.1	Size of the containers were the same as the cloth-lining workshop, the number was a half of that workshop	1.0
3.1	East assembly	assembly	181.4	Size of the only container was that of the cloth-lining workshop	1.2
3.2	East assistant	vulcanizing, cutting cloth	181.4	Indirect exposure to the East Assembly	5.0
4.1	Steel weaving	steel weaving	120.3	Indirect exposure to Steel Weaving Assembly	2.0
4.2	Steel weaving assembly	steel weaving assembly	120.3	Sources were tubes of 2.5 cm radius and 25 cm length, surface was about 478 cm^2^ (about 1/15 of the cloth-lining workshop)	1.0
4.3	Steel weaving assistant	vulcanizing,, wrapping cloth	120.3	Indirect exposure to Steel Weaving Assembly	4.0
5.1	Line 3 assistant	feeding rubber mud	180.5	Indirect exposure to Line 3 Assembly	3.0
5.2	Line 3 assembly	assembly	180.5	6 assembly machines, 2 times of the steel weaving assembly	1.0
6.1	Line 1–2	assembly, feeding materials, cutting tubes	79.6	1/3 of the line 3	2.0
7.1	Slurry applying	slurry	17.2	2 square boxes of 75×15 cm (surface about 2261 cm^2^)	1.2
7.2	Cotton tube weaving	applying weaving and applying slurring with machine	26.5	15 slurry boxes of 50×25 cm	4.5
7.3	Cotton tube assistant	cutting cloth, staving tapes, vulcanizing	26.5	Indirect exposure to Cotton Tube Weaving	6.4
8.1	Cloth-lining assembly	cloth-lining assembly	95.7	10 containers of 20×30 cm(volatile surface about 6,000 cm^2^)	1.8
8.2	Cloth-lining assistant	cutting cloth, staving tapes, vulcanizing	62.9	Indirect exposure to Cloth-lining Assembly	4.0

**Table 3.** Priors provided by the experts on the parameters of the Model.

Code	Workstation	Pollutant production rate [AM(CI 90%), mg/min]	Workshop air flow [GM(CI 90%), m^3^/min]	Interface air flow [GM(CI 90%), m^3^/min]
1.1	Slurry making	200(100–300)	154(31–772)	5(1–27)
2.1	Core assembly	826(413–1239)	735(147–3675)	22(4–109)
3.1	East assembly	165(83–248)	6634(1327–33171)	31(6–156)
3.2	East assistant	165(83–248)	6634(1327–33171)	539(108–2696)
4.1	Steel weaving	110(55–165)	4403(881–22014)	86(17–432)
4.2	Steel weaving assembly	110(55–165)	4403(881–22014)	22(4–109)
4.3	Steel weaving assistant	110(55–165)	4403(881–22014)	347(69–1736)
5.1	Line 3 assistant	220(110–330)	6634(1327–33171)	194(39–972)
5.2	Line 3 assembly	220(110–330)	6634(1327–33171)	22(4–109)
6.1	Line 1–2	73.2(37–110)	2922(584–14610)	86(17–432)
7.1	Slurry applying	520(260–780)	629(126–3145)	25(5–125)
7.2	Cotton tube weaving	4400(2200–6600)	973(195–4863)	437(87–2185)
7.3	Cotton tube assistant	4400(2200–6600)	973(187–4685)	874(175–4370)
8.1	Cloth-lining assembly	1302(651–1953)	3501(700–17505)	71(14–355)
8.2	Cloth-lining assistant	1652(826–2478)	2298(460–11492)	347(69–1736)

**Table 4.** Area concentrations (CI 95%, mg/m^3^) of airborne benzene by field surveying, historic monitoring and Bayesian estimating.

Code	Workstation	Sampling day	Field Surveying		Historic Monitoring		Bayesian Estimating
			Sample number (<LOD)	Geometric mean (CI 95%)	Sample number (<LOD)	Geometric mean (CI 95%)	Geometric mean (CI 95%)
1.1	Slurry making				17 (1)	152.2 (8.7–2662.3) −	111.5 (46.6–266.6)
2.1	Core assembly				12 (1)	1.245 (0.1–18.9) −	1.8 (1.4–2.5)
3.1#	East assembly	10	20 (3)	3.0 (1.1–8.5)−	11 (1)	4.5 (1.1–18.9)−	6.1 (1.2–30.1)
3.2	East assistant				11 (2)	0.9 (0.0–22.9)	0.5 (0.4–0.6)
4.1	Steel weaving				10 (2)	20.7 (0.2–1958.0) −	3.7 (2.1–6.6)
4.2	Steel weaving assembly				13 (2)	61.2 (1.6–2411.4)	19.2 (15.1–24.4)
4.3#	Steel weaving assistant	10	20 (5)	0.7 (0.1–6.3) +	6 (1)	1.3 (0.1–14.6) −,+	1.1 (0.6–1.9)
5.1	Line 3 assistant				6 (1)	4.8 (2.9–8.1)	3.3 (1.4–7.6)
5.2#	Line 3 assembly	10	20 (2)	17.3 (3.5–86.4) −	5 (1)	40.6 (20.4–80.7)	21.4 (15.9–28.8)
6.1#	Line 1–2	10	20 (7)	0.5 (0.1–2.7)	4 (1)	2.0 (0.4–11.4) −	0.9 (0.1–6.6)
7.1#	Slurry applying	9	18 (3)	69.8 (62.2–78.2) −	12 (1)	60.9 (2.4–1573.2) −	41.6 (13.5–128.9)
7.2	Cotton hose weaving				7 (1)	2.4 (0.4–14.4) −	0.9 (0.6–1.6)
7.3	Cotton hose assistant				8 (2)	6.2 (1.5–26.2)	10.9 (3.0–39.5)
8.1#	Cloth-lining assembly	9	18 (2)	89.5 (34.8–230.5) −	1 (0)	409.4**	125.7 (18.9–837.5)
8.2#	Cloth-lining assistant	10	20 (2)	16.8 (2.5–114.) −	1 (0)	51	11. 9 (3.3–43.3)

+: normal distribution test, p<0.05;

−: log-normal distribution test, p<0.05

#: workstation where field samples taken

** p<0.01;

* p<0.05, compared with field sampling results

## References

[1] Semple S (2006). Assessing occupational and environmental exposure. Occup. Med.

[2] Esmen N (1979). Retrospective industrial hygiene surveys. Am. Ind. Hyg. Assoc. J.

[3] Cherrie JW, Schneider T (1999). Validation of a new method for structured subjective assessment of past concentrations. Ann. Occup. Hyg.

[4] Burstyn I, Kromhout H (2002). A critique of Bayesian methods for retrospective exposure assessment. Ann. Occup. Hyg.

[5] Gurumurthy R, James HV (1999). A Bayesian approach to retrospective exposure assessment. Appl. Occup. Envir. Hyg.

[6] Gurumurthy R (2001). Retrospective exposure assessment using Bayesian methods. Ann. Occup. Hyg.

[7] Spiegelhalter DJ, Myles JP, Jones DR, Abrams KR (2004). Bayesian methods in health technology assessment: a review. Health Tech. Assess.

[8] Burstyn I, Teschke K (1999). Studying the determinants of exposure: a review of methods. Am. Ind. Hyg. Assoc. J.

[9] Lunn DJ, Thomas A, Best N, Spiegelhalter D (2000). WinBUGS--a Bayesian modelling framework: concepts, structure, and extensibility. Statistic. Comput.

[10] Fong Y, Wang L (1992). Maximum Allowable Concentration in the air of the workplace. Ind. Hyg. Occup. Dis.

[11] Corn M, Esmen NA (1979). Workplace exposure zones for classification of employee exposures to physical and chemical agents. Am. Ind. Hyg. Assoc. J.

[12] Burdorf A, Tongeren M (2003). Commentary: Variability in workplace exposures and the design of efficient measurement and control strategies. Ann. Occup. Hyg.

[13] Collins JJ, Ireland BK, Easterday PA, Nair RS, Braun J (1979). Evaluation of lymphopenia among workers with low-level benzene exposure and the utility of routine data collection. Occup. Environ. Med.

[14] Charles BK (2000). Two-Zone Steady State Model. In Mathematical models for estimating occupational exposure to chemicals.

[15] Shi M, Zhang M, Xu Y, Zhu Y (2005). Experimental research on gas Chromatographic determination for o-chlorotoluene collected by activated charcoal in the workplace air. J. Environ. Occup. Med.

[16] Greenland S (2006). Bayesian perspectives for epidemiological research: I. foundations and basic methods. Int. J. Epidemiol.

[17] Hornung RW, Reed LD (1990). Estimation of average concentration in the presence of nondetectable values. Appl. Occup. Environ. Hyg.

[18] Bohong X, Huifang Y (2003). Method of Sampling and Analysis in Workplace.

[19] Li Y (2000). Determination of benzene in the atmosphere of workshop by chromatography. Guizhou Chem. Ind.

[20] Money CD, Margary SA (2002). Improved use of workplace exposure data in the regulatory risk assessment of chemicals within Europe. Ann. Occup. Hyg.

[21] Vocht F, Straif K, Szeszenia-Dabrowaka, Hagmar L, Tom S, Burstyn I, Vermeulen R, Kromhout H (2005). A database of exposures in the rubber manufacturing industry: design and quality control. Ann. Occup. Hyg.

[22] Astrakianakis G, Seixas SN, Camp JE, Christiani CD, Feng Z, Thomas BD, Checkoway H (2006). Modeling, estimation and validation of cotton dust and endotoxin exposures in Chinese textile operations. Ann. Occup. Hyg.

[23] Lavoué J, Bégin D, Beaudry C, Gérin M (2007). Monte Carlo simulation to reconstruct formaldehyde exposure levels from summary parameters reported in the literature. Ann. Occup. Hyg.

